# Supplementary material for: Endoplasmic Reticulum Protein Disulfide Isomerase Shapes T Cell Efficacy for Adoptive Cellular Therapy of Tumors
Source: Cells. 2019 Nov 26;8(12):1514. doi: 10.3390/cells8121514 (PMC6953024; doi:10.3390/cells8121514)
Supplement: Supplementary file 1 [file cells-08-01514-s001.pdf]

Supplemental Figure 1. Dose Curve Viability of E64FC26 in Healthy T Cells

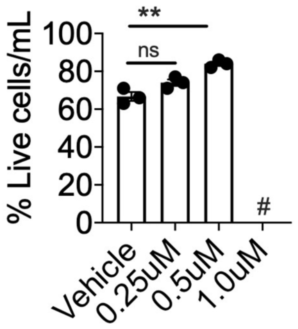

Supplemental Figure 1. Dose Curve Viability of E64FC26 in Healthy T Cells. Pmel T cells activated with gp100 peptide and expanded in the presence of vehicle or 0.25 µM, 0.5 µM PDI inhibitor E64FC26 for 3 days then split into fresh drug and media and expanded for 4 more days. #, T cells activated in 1.0 µM E64FC26 were not expanded after 3 days of activation due to low cell yield.

Supplemental Figure 2. Glycolytic Capacity of E64FC26-Treated T Cells.

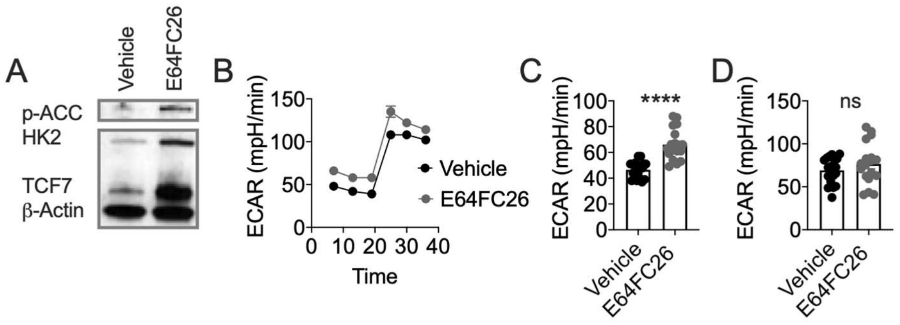

Supplemental Figure 2. Glycolytic Capacity of E64FC26-Treated T Cells. Pmel T cells activated with gp100 peptide and expanded in the presence of vehicle or E64FC26. A) Western blotting for indicated proteins with β-actin as loading control. B) Extracellular acidification rate (ECAR) trace and C) quantification of C) basal ECAR rates and D) glycolytic capacity in response to oligomycin addition. Individual experiment performed 3 times. C-D, Student's t test, ****p<0.0001, ns = not significant.
